# Postural Balance and Human Movement: An Integrative Framework for Mechanisms, Assessment, and Functional Implications

**DOI:** 10.3390/jcm15072588

**Published:** 2026-03-28

**Authors:** Eduardo Guzmán-Muñoz, Felipe Montalva-Valenzuela, Exal Garcia-Carrillo, Antonio Castillo-Paredes, José Francisco López-Gil, Jose Jairo Narrea Vargas, Rodrigo Yáñez-Sepúlveda, Yeny Concha-Cisternas

**Affiliations:** 1 Escuela de Kinesiología, Facultad de Salud, Universidad Santo Tomás, Talca 3460000, Chile; eguzmanm@santotomas.cl (E.G.-M.); yenyconchaci@santotomas.cl (Y.C.-C.); 2 Escuela de Pedagogía en Educación Física, Facultad de Educación, Universidad Autónoma de Chile, Talca 3460000, Chile; 3 Escuela de Entrenador en Actividad Física y Deporte, Facultad de Ciencias Humanas, Universidad Bernardo O’Higgins, Santiago 8370040, Chile; 4 Department of Physical Activity Sciences, Faculty of Education Sciences, Universidad Católica del Maule, Talca 3480112, Chile; 5 Department of Physical Activity Sciences, Universidad de Los Lagos, Osorno 5290000, Chile; 6 Grupo AFySE, Investigación en Actividad Física y Salud Escolar, Escuela de Pedagogía en Educación Física, Facultad de Educación, Universidad de Las Américas, Santiago 8370040, Chile; acastillop85@gmail.com; 7 School of Medicine, Universidad Espíritu Santo, Samborondón 092301, Ecuador; 8 Department of Sport Sciences, Faculty of Sport and Health Sciences, Fit Generation Research Institute, AD500 Andorra la Vella, Andorra; 9 Facultad de Ciencias de la Salud, Carrera de Nutrición y Dietética, Universidad San Ignacio de Loyola, Lima 15024, Peru; jose.narrea@usil.pe; 10 Faculty Education and Social Sciences, Universidad Andrés Bello, Viña del Mar 2520000, Chile; rodrigo.yanez.s@unab.cl; 11 Vicerrectoría de Investigación e Innovación, Universidad Arturo Prat, Iquique 1100000, Chile

**Keywords:** postural balance, postural control, multisensory integration, sensory reweighting, center of pressure, clinical balance tests, falls, motor control, athletes, pediatrics

## Abstract

Postural balance is a foundational component of human motor behavior, yet it remains conceptually ambiguous and methodologically heterogeneous across the clinical, educational, and sport sciences. This narrative review aims to provide an integrative framework that clarifies key concepts (postural control vs. postural balance), synthesizes the main sensorimotor and biomechanical mechanisms underpinning balance, and organizes current assessment approaches and functional implications across populations. Narrative literature synthesis was conducted to integrate evidence covering multisensory integration and sensory reweighting, central neural control (spinal, brainstem, cerebellar, and cortical contributions), neuromuscular and biomechanical strategies (e.g., ankle/hip/stepping), and cognitive influences (e.g., dual-task effects). We further summarize commonly used instrumental outcomes derived from force-platform center-of-pressure metrics and widely adopted clinical and functional balance tests, highlighting their typical applications and limitations across the lifespan including pediatric, general adults, older adults, and athletic populations. This review proposes a closed-loop, systems-based model in which postural balance is conceptualized as an emergent functional outcome arising from distributed postural control processes shaped by task, environmental, and individual constraints. In conclusion, integrating mechanistic understanding with population-specific assessment enhances interpretability and supports more precise, context-sensitive balance evaluation and intervention in both health and performance settings.

## 1. Introduction

Postural balance is a foundational component of human motor behavior, enabling the body to maintain stability while interacting dynamically with the environment [1,2]. Historically, viewed through a reflex-based lens, postural control was once considered a set of automatic adjustments aimed at counteracting gravity and maintaining an upright stance. However, contemporary perspectives recognize postural balance as a complex and adaptive motor skill that reflects the dynamic integration of sensory inputs, neural control processes, and biomechanical mechanisms across multiple systems [3,4]. This integrated capacity not only supports the maintenance of an upright posture but also underpins the execution of nearly all voluntary movements, from simple tasks of daily living to high-performance athletic activities.

As research on postural balance has expanded, so has recognition of its multidimensional nature. Postural control involves the coordination of anticipatory and reactive strategies, the continuous weighting of multisensory information, and the modulation of neuromuscular responses according to task and environmental demands [3,5]. These processes are further shaped by individual factors such as age, health status, physical condition, and cognitive function [6,7,8,9]. Consequently, balance cannot be understood as a fixed attribute but rather as a flexible skill that varies across contexts and populations.

Despite its fundamental importance, postural balance remains a challenging construct to define, assess, and train. The literature is marked by conceptual ambiguity, particularly in the interchangeable use of terms such as “postural control” and “balance”, and methodological heterogeneity in how balance is evaluated. Furthermore, much of the research is siloed within specific disciplines (e.g., neurology, sports science, and geriatrics), limiting the development of integrative models that bridge theory, assessment, and practice. The purpose of this narrative review is to offer a comprehensive and integrative framework for understanding postural balance as a prerequisite for human movement.

## 2. Methods

This study was conducted as a structured narrative review aimed at integrating and synthesizing the conceptual, physiological, and functional dimensions of postural balance. The objective of the review was not to estimate pooled quantitative effects but rather to provide an integrative framework that organizes current knowledge regarding the mechanisms underlying postural balance, its assessment approaches, and its functional implications across different populations.

The literature search was performed between October and December 2025 using three major electronic databases: PubMed, Scopus, and Web of Science. The search strategy was designed to identify studies addressing the conceptualization, mechanisms, and evaluation of postural balance and postural control in humans. Search terms included combinations of keywords such as “postural balance”, “postural control”, “postural stability”, “multisensory integration”, “sensory reweighting”, “center of pressure”, “posturography”, and “balance assessment”. Boolean operators (AND and OR) were used to combine the terms, and the search syntax was adapted to the requirements of each database. In addition to the database search, the reference lists of relevant articles and reviews were manually screened in order to identify additional publications that could contribute to the conceptual and methodological framework of the review.

Studies were considered eligible if they addressed human postural balance or postural control and examined sensorimotor, neural, biomechanical, or functional aspects related to balance regulation. Both original research articles and review papers published in peer-reviewed scientific journals were considered when they contributed to the theoretical understanding, methodological approaches, or functional implications of postural balance. Studies focusing exclusively on animal models, those unrelated to postural balance, or publications not available in English were excluded. Titles and abstracts identified through the search process were initially screened to determine potential relevance, after which full texts of the selected articles were reviewed to confirm their eligibility.

The selected literature was analyzed with the aim of identifying recurring conceptual themes and methodological approaches related to postural balance. Particular attention was given to the definitions of postural balance and postural control, the sensorimotor mechanisms underlying balance regulation, the instrumental and clinical methods used to assess balance, and the functional implications of balance across different populations and contexts. The evidence was synthesized narratively to develop an integrative conceptual framework linking theoretical mechanisms, assessment strategies, and applied implications in health, rehabilitation, and performance settings.

## 3. Conceptual and Theoretical Framework of Postural Balance

### 3.1. Postural Control and Postural Balance: Conceptual Distinctions and Evolution of Perspectives

The study of postural balance has undergone substantial conceptual evolution over recent decades. Early approaches to posture and balance were largely grounded in a reflex-based paradigm in which postural control was understood as a set of automatic righting and equilibrium reflexes, predominantly mediated by subcortical structures and primarily aimed at counteracting the effects of gravity [10]. Within this framework, balance was viewed as the outcome of stereotyped responses to external perturbations, with limited consideration of task demands, environmental constraints, or individual variability.

Contemporary perspectives have largely moved beyond this reductionist view. Current evidence supports the notion that postural control represents a complex sensorimotor process through which the central nervous system continuously integrates multisensory information and organizes anticipatory and reactive motor responses to regulate body orientation and stability in space [1,11,12]. From this standpoint, postural control refers to the underlying regulatory mechanism—neural, sensory, and neuromuscular—that govern how posture is maintained and adjusted across different conditions.

Within this conceptual framework, postural balance can be more precisely defined as a complex motor skill that emerges from the effectiveness of postural control processes [13,14]. Postural balance reflects the functional ability to maintain or restore stability by controlling the position and motion of the body’s center of mass relative to the base of support under both static and dynamic conditions [4,13,14]. Importantly, balance should not be considered an independent system but rather an observable and measurable expression of how efficiently postural control mechanisms operate in a given context.

Although the distinction between postural control as a regulatory process and postural balance as its functional outcome has been widely articulated in the literature [1,2,13]. This review builds upon that conceptual foundation to propose an integrative perspective. Specifically, we conceptualize postural balance as an emergent functional outcome arising from distributed postural control processes operating within task, environmental, and individual constraints.

This close relationship between process and outcome helps explain why the terms postural control and postural balance are often used interchangeably in both research and clinical practice. While postural control emphasizes the mechanisms responsible for regulating posture, postural balance highlights the functional manifestation of those mechanisms as a motor capability. Conceptually, however, distinguishing between these terms allows for a clearer understanding of how neural regulation translates into functional motor performance.

A key theoretical contribution to this field has been the identification of the two primary functional goals of postural control: postural orientation and postural stability. Postural orientation refers to the ability to maintain an appropriate alignment between body segments, as well as between the body and the surrounding environment, particularly with respect to the gravitational vertical [2]. Postural stability, on the other hand, refers to the capacity to control the center of mass within the limits of the base of support, thereby preventing loss of balance [2]. These two objectives operate simultaneously and synergistically, and their integration is essential for the effective expression of postural balance as a motor skill.

The evolution from a reflex-based interpretation toward this integrative perspective has led to the recognition that postural balance is not a passive or purely reactive condition. Rather, it is an active, adaptive, and task-dependent function in which the central nervous system continuously selects and modulates control strategies in response to mechanical, sensory, and cognitive demands.

### 3.2. Postural Balance as a Prerequisite for Human Movement

In addition to its role in maintaining an upright stance, postural balance should be understood as a functional prerequisite for human movement [15,16]. Virtually all voluntary motor actions, from basic activities such as standing up, reaching, or walking, to complex athletic movements, require adequate postural balance to be executed effectively and safely [13,15,16].

From a motor control perspective, this relationship is largely mediated by anticipatory postural adjustments (APAs), which occur prior to the onset of voluntary movement [17,18]. These adjustments serve to counteract the internally generated perturbations associated with movement by activating stabilizing muscle synergies in advance, thereby preserving balance [17,18]. In this sense, postural balance does not simply respond to movement; it precedes and organizes it, creating the conditions necessary for efficient motor execution.

During dynamic tasks, postural balance assumes a continuous and adaptive role. Activities such as gait, running, or directional changes involve repeated transitions through states of controlled instability, in which the center of mass is deliberately displaced and recovered [19]. Effective balance during these tasks depends on the coordinated interaction between anticipatory mechanisms and reactive responses, allowing the individual to remain stable while moving [20,21].

When postural balance is compromised, movement quality is markedly affected. Deficits in balance are associated with increased muscular co-contraction, reduced movement velocity and accuracy, and the emergence of compensatory strategies that increase energetic cost and injury risk [22,23]. Thus, postural balance does not determine whether movement is possible but rather how movement is performed, shaping its efficiency, safety, and adaptability. This distinction underscores the central role of balance as a foundational element of functional motor behavior.

### 3.3. Task, Environmental, and Individual Constraints on Postural Balance

An essential aspect of contemporary postural balance theory is the recognition that balance performance is highly context-dependent. Postural balance is not a fixed or generalized capacity but rather an adaptive function that emerges from the interaction between the individual, the task, and the environment [24,25].

Task characteristics, such as movement complexity, speed requirements, the base of support, and the need for dual-task processing, directly influence the postural strategies employed [26]. As task demands increase, greater reliance is placed on anticipatory control, multisensory integration, and higher-level neural resources [27,28]. Similarly, environmental conditions introduce variability in sensory and mechanical constraints, requiring continuous sensory reweighting and strategy adaptation [27]. Factors such as unstable surfaces, reduced visual input, or sensory conflict significantly alter postural behavior [29,30].

Moreover, individual constraints, including age, motor experience, health status, body composition, and physical fitness, shape the capacity to integrate sensory information and generate effective postural responses [8,31,32]. These constraints help explain why identical postural challenges can elicit markedly different balance behaviors across individuals or populations.

Taken together, these interactions reinforce the view of postural balance as a flexible and context-sensitive motor skill arising from the dynamic coupling of sensory systems, neural control processes, and motor execution. This conceptual framework provides a critical foundation for interpreting balance assessments and for understanding the functional implications of postural balance across clinical, athletic, and educational settings.

Although foundational models such as Horak’s systems model of postural control have been instrumental in identifying the multiple subsystems involved in balance regulation, the present framework seeks to extend this perspective by explicitly integrating three dimensions that are often addressed separately in the literature: the underlying sensorimotor mechanisms of postural control, the assessment strategies used to evaluate balance, and the functional manifestations of balance across different populations and contexts. Within this perspective, postural balance is conceptualized as an emergent functional outcome arising from distributed postural control processes operating under task, environmental, and individual constraints. By linking mechanistic understanding with commonly used assessment approaches and population-specific implications, the framework aims to provide a clearer conceptual bridge between theoretical models of postural control and their practical interpretation in clinical, developmental, and sport settings.

## 4. Sensorimotor Mechanisms Underlying Postural Balance

**Figure 1:**
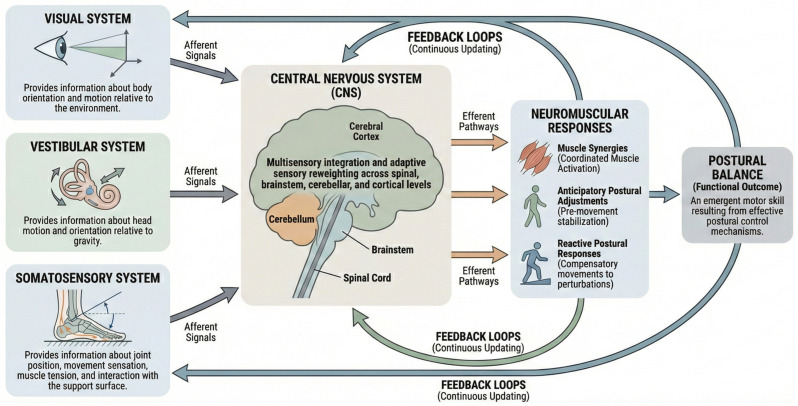
Conceptual schematic summarizing the sensorimotor regulation of postural balance within a closed-loop control framework. The diagram integrates established principles from the motor control literature, illustrating the interaction between sensory inputs, central neural integration, and neuromuscular responses that collectively contribute to postural balance. The figure provides an integrative visualization of mechanisms discussed throughout the review. Postural balance emerges from the continuous interaction between sensory inputs, central integration, and neuromuscular responses. The visual, vestibular, and somatosensory systems provide complementary afferent information regarding body orientation, motion, and interaction with the support surface. These signals are integrated across multiple levels of the central nervous system, including the spinal cord, brainstem, cerebellum, and cerebral cortex, through parallel and distributed processing mechanisms involving multisensory integration and adaptive sensory reweighting. Central neural processing generates coordinated neuromuscular responses, including muscle synergies, anticipatory postural adjustments, and reactive postural responses, which contribute to the regulation of posture under both predictable and unpredictable conditions. The resulting motor output continuously modifies sensory input, generating updated afferent feedback that sustains closed-loop regulation of posture. Within this framework, postural balance is conceptualized as an emergent functional outcome arising from the effective operation of postural control mechanisms.

Postural balance emerges from the continuous interaction of multiple sensorimotor mechanisms that allow the body to maintain stability while adapting to internal and external perturbations [1,11]. These mechanisms operate across different levels of the nervous system and involve the integration of sensory inputs, central processing, and coordinated neuromuscular responses [11,30]. Understanding these mechanisms is essential for interpreting balance behavior, both in static and dynamic conditions, and for explaining why balance performance varies across tasks, environments, and populations (Figure 1).

### 4.1. Sensory Systems and Sensory Reweighting

Postural control relies on the integration of sensory information from three primary sources: the visual, vestibular, and somatosensory systems [11]. The main characteristics and functional roles of these sensory systems in postural control and their implications for postural balance are summarized in Table 1. Each system provides complementary information about body orientation, motion, and interaction with the environment, and their relative contribution to balance regulation is dynamically adjusted according to task demands and sensory availability.

The visual system supplies information regarding the position and movement of the body relative to the environment, contributing strongly to the perception of verticality and self-motion [33,34]. Visual input is particularly influential during quiet stances and slow movements; the removal or degradation of visual information (e.g., eyes closed or visual conflict) consistently results in increased postural sway [35,36], highlighting its stabilizing role.

The vestibular system provides information about head motion and orientation relative to gravity through the semicircular canals and otolith organs [37,38]. Vestibular input is critical when visual or somatosensory information is unreliable, and it plays a central role in maintaining balance during head movements and in dynamic tasks that involve changes in acceleration [37,38]. Unlike visual and somatosensory signals, vestibular input inherently references gravity, making it indispensable for postural orientation.

The somatosensory system encompasses proprioceptive and cutaneous receptors located in muscles, tendons, ligaments, joints, and the plantar surface of the feet [39]. Specifically, the proprioceptive system provides information about joint position, movement sensation, and muscle tension [39]. In an upright stance, ankle proprioception and plantar cutaneous feedback are particularly important for detecting small deviations in the center of mass and initiating corrective responses.

A key feature of postural control is sensory reweighting, which is defined as the adaptive process by which the central nervous system dynamically adjusts the relative contribution of sensory inputs on the basis of their reliability and relevance [27]. For example, when standing on an unstable surface, somatosensory input becomes less reliable, and greater reliance is placed on visual and vestibular cues. Sensory reweighting allows balance to be maintained across a wide range of environmental conditions but can be compromised in aging, neurological disorders, or musculoskeletal injury, leading to impaired postural balance [27].

### 4.2. Central Integration and Neural Control of Posture

Sensory information related to posture is integrated across multiple levels of the central nervous system, including the spinal cord, brainstem, cerebellum, and cerebral cortex [40,41]. Rather than functioning hierarchically, these structures operate in parallel, contributing to different aspects of postural regulation [40].

At the brainstem and spinal level, relatively fast and automatic postural responses are generated to counteract sudden perturbations [42]. These responses are organized into coordinated muscle synergies that act to stabilize the body efficiently. The cerebellum plays a crucial role in calibrating these responses by integrating sensory feedback, predicting the consequences of movement, and refining motor output to ensure accuracy and adaptability [41].

Cortical structures contribute to postural control, particularly during voluntary movements, complex tasks, or situations involving uncertainty or dual-task demands [43]. Cortical involvement is evident in anticipatory postural adjustments, in the adaptation of balance strategies through learning, and in conditions requiring the conscious control of posture [44,45]. As task complexity increases, greater cortical resources are recruited, which helps explain the interaction between balance performance and cognitive load.

Importantly, postural control involves both feedforward and feedback mechanisms. Feedforward control, expressed through anticipatory postural adjustments, prepares the body for expected perturbations associated with voluntary movement [14,46,47]. In contrast, feedback control relies on sensory input to generate corrective responses after a perturbation has occurred [14,46,47]. Effective postural balance depends on the coordinated interaction between these mechanisms, allowing stability to be preserved under both predictable and unpredictable conditions [14,46,47].

### 4.3. Neuromuscular and Biomechanical Mechanisms

At the neuromuscular level, postural balance is supported by the coordinated activation of muscles that generate forces to counteract gravity and perturbations. A baseline level of muscle activity, often referred to as postural tone, contributes to the intrinsic stiffness of the musculoskeletal system and provides passive resistance to small disturbances [48]. This intrinsic stiffness reduces the need for large corrective actions and enhances postural stability during quiet stances [49,50].

When perturbations exceed the capacity of intrinsic stiffness, active neuromuscular responses are recruited through well-established postural strategies, classically described as the ankle, hip, and stepping strategies [1]. The ankle strategy predominates during small perturbations on firm surfaces and involves distal-to-proximal muscle activation patterns to restore balance [51,52]. The hip strategy is employed during larger or faster perturbations or when the base of support is reduced, generating rapid movements of the trunk and pelvis to reposition the center of mass [1]. When stability cannot be restored through these strategies, a stepping response is initiated to realign the base of support with the displaced center of mass [1].

Biomechanically, postural balance reflects the continuous regulation of the relationship between the center of mass and the base of support, achieved through adjustments in joint torques and muscle forces [53,54]. Differences between anteroposterior and mediolateral control further illustrate the complexity of balance regulation, as mediolateral stability relies more heavily on the hip and trunk musculature and is particularly sensitive to age-related decline and pathology.

Together, these neuromuscular and biomechanical mechanisms provide the physical means through which neural control processes are translated into stable and adaptable postural behavior.

### 4.4. Cognitive Influences on Postural Balance

Although often considered automatic, postural balance is influenced by cognitive processes, particularly in challenging or novel situations. Attention, executive function, and working memory contribute to balance regulation when sensory information is ambiguous or when simultaneous tasks are performed.

Dual-task paradigms have demonstrated that performing a cognitive task while maintaining balance can degrade postural performance, especially in older adults and clinical populations [55,56]. This interaction suggests that postural balance competes for shared neural resources and that cognitive decline or increased attentional demands can compromise balance control.

The involvement of cognitive processes further supports the view of postural balance as a high-level motor skill rather than a purely reflexive function [57]. This study also highlights the importance of considering cognitive–motor interactions when assessing balance and designing interventions aimed at improving functional stability.

The mechanistic domains described in this section are closely reflected in the contexts in which postural balance is assessed. For instance, processes such as sensory reweighting become particularly evident under sensory manipulation conditions frequently used in posturographic protocols, including altered visual input or unstable support surfaces. Similarly, anticipatory postural adjustments are especially relevant in dynamic tasks that involve voluntary movement initiation, such as gait initiation, reaching tasks, or transitional movements assessed in functional balance tests. These relationships illustrate how the underlying mechanisms of postural control are not only theoretical constructs but can also be indirectly observed through commonly used instrumental and clinical balance assessments. Understanding these links provides an important foundation for interpreting balance performance across different tasks, environments, and populations.

## 5. Assessment of Postural Balance

The assessment of postural balance represents a critical link between theoretical understanding and practical application. Given that postural balance is a complex motor skill emerging from multiple control processes, its evaluation requires tools capable of capturing different dimensions of stability, orientation, and functional performance. However, the absence of a universal gold standard has led to substantial heterogeneity in assessment approaches, ranging from laboratory-based instrumental measures to clinical and functional tests. Understanding what each method captures, and its limitations, is essential for meaningful interpretation of balance outcomes.

### 5.1. Instrumental Assessment of Postural Balance

**Figure 2:**
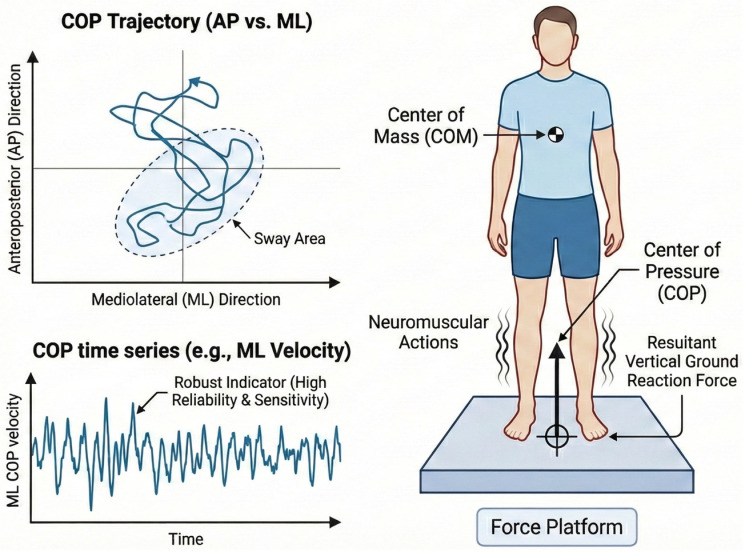
Center of pressure (COP) trajectory during static postural assessment using a force platform. Schematic representation of center of pressure (COP) behavior during quiet standing assessed with a force platform. The (**left**) panel illustrates the COP trajectory in the anteroposterior (AP) and mediolateral (ML) directions, where the blue line represents the continuous displacement of the COP over time, and the spatial dispersion of the trajectory reflects the postural sway area (dashed blue region). The (**lower**) panel depicts the COP mediolateral velocity time series, where the blue line represents the temporal fluctuations of COP velocity, which is considered a robust and sensitive indicator of postural control because of its high reliability and responsiveness to neuromuscular adjustments. The (**right**) panel shows the biomechanical model underlying the COP measurement, including the relationship between the center of mass (COM), neuromuscular actions, and the resultant vertical ground reaction force recorded by the force platform. COP displacement emerges from continuous neuromuscular corrections that regulate body stability. Together, spatial (trajectory) and temporal (velocity) COP metrics provide complementary information for quantifying postural control performance and detecting subtle balance impairments.

Laboratory-based assessments of postural balance are most commonly performed via force platforms, which quantify postural sway through measurements of the center of pressure (COP) [58,59]. The COP represents the point of application of the resultant vertical ground reaction force and reflects the neuromuscular actions used to control the position of the center of mass relative to the base of support (Figure 2) [4,58].

From COP trajectories, a wide range of variables can be derived, including displacement, velocity, root mean square (RMS), and sway area, which are typically analyzed in the anteroposterior and mediolateral directions [4,58]. Among these metrics, COP velocity has consistently demonstrated greater reliability and sensitivity to change than spatial measures such as sway amplitude or area, particularly during quiet standing tasks [60,61,62]. Consequently, COP velocity is often considered one of the most robust indicators of postural balance performance.

Despite their objectivity and high temporal resolution, COP-based measures are not without limitations. COP variables do not directly represent the motion of the center of mass but rather the corrective forces generated to control it [63,64]. As such, greater COP excursions may reflect either impaired balance control or adaptive exploratory behavior, depending on task demands and individual strategies. This ambiguity highlights the importance of contextual interpretation rather than assuming a linear relationship between increased sway and poorer balance.

Methodological factors, including sampling frequency, trial duration, foot position, visual conditions, and surface characteristics, strongly influence COP outcomes and must be carefully standardized [65,66]. Inconsistent protocols across studies contribute to variability in reported results and limit comparability. Therefore, instrumental assessments provide valuable insight into postural control mechanisms, but their interpretation requires a clear understanding of task constraints and measurement assumptions.

### 5.2. Clinical and Functional Assessment of Postural Balance

In contrast to laboratory-based methods, clinical and functional balance assessments emphasize an individual’s ability to maintain postural stability during task-oriented and functionally meaningful activities. These assessments are widely used in clinical, rehabilitation, educational, and sport settings because of their feasibility, low cost, and strong ecological validity. Importantly, the selection of a clinical balance test varies according to age, functional capacity, and specific motor demands characteristic of each population (Table 2).

In older adults, the most frequently used clinical tools include the Berg balance scale, the timed up and go (TUG) test, the Tinetti performance-oriented mobility assessment, and single-leg stance tests [67,68]. These instruments are strongly associated with mobility limitations, fall risk, and loss of functional independence [67,69]. They assess postural balance during everyday tasks such as transfers, gait initiation, turning, and quiet standing, making them particularly relevant for geriatric assessment and fall prevention programs. While widely adopted, these tools may exhibit ceiling effects in high-functioning older adults and limited sensitivity to subtle balance impairments.

In pediatric populations, clinical balance assessment must be adapted not only to the child’s chronological age but also to developmental stage, cognitive–motor maturity, and task demands [70]. In preschool-aged and early school-aged children, balance is most commonly evaluated via simple and age-appropriate tasks that target static body stability and quasimobility, such as single-leg stances, tandem stances, and observational or scale-based instruments, including the Pediatric Balance Scale [70,71,72]. These measures primarily assess basic postural stability and emerging sensorimotor control and are appropriate for early stages of motor development, when attentional capacity and task comprehension are still maturing.

As children grow older, balance assessment progressively incorporates tasks that challenge postural control under more demanding task constraints. In school-aged children and adolescents, clinical evaluation frequently includes dynamic tasks with a reduced base of support or controlled locomotion, such as tandem walking, balance beam walking, or line walking, often derived from standardized motor proficiency batteries (e.g., BOT-2) [73,74]. These tasks assess dynamic body stability and stability during locomotion rather than simple static balance.

In adolescents with sufficient motor competence, particularly in physically active or sport-oriented contexts, dynamic unipedal reach tasks such as the Star Excursion Balance Test (SEBT) or its standardized derivative, the Y-Balance Test, may be applied to evaluate dynamic postural balance [4,75]. However, these tests are not routinely used in general pediatric clinical assessment and are primarily indicated when higher-level postural demands are relevant.

In the general adult population, assessment strategies depend largely on an individual’s functional status. For sedentary or average adults, postural balance is typically assessed via simple static and quasidynamic tests, such as single-leg stance (eyes open and closed), tandem stance, and the functional reach test. However, since these measures often exhibit ceiling effects in healthy, active adults, more challenging dynamic tasks are required to detect subtle deficits. Consequently, instruments typically reserved for athletes, such as the SEBT or the Y-Balance Test, are increasingly applied in the active general population to evaluate dynamic postural balance under conditions that demand greater strength, range of motion, and neuromuscular integration [76,77].

In athletic populations, dynamic balance tests predominate, particularly the SEBT and Y-Balance Test, owing to their ability to challenge postural control under conditions that closely resemble sport-specific demands [78,79,80,81]. These tools are widely used for injury risk screening, monitoring rehabilitation progress, and informing return-to-play decisions, especially in sports involving jumping, cutting, or rapid changes in direction.

Despite their clinical utility, functional balance tests provide limited insight into the specific sensorimotor mechanisms underlying postural control. Performance outcomes typically reflect the combined influence of strength, coordination, reaction time, and cognitive processing, making it difficult to isolate deficits within individual sensory, neuromuscular, or neural subsystems. Moreover, ceiling effects are common in young, healthy, or highly trained individuals, reducing sensitivity to subtle impairments.

Nevertheless, clinical and functional assessments remain indispensable because of their strong associations with real-world performance and functional outcomes. Their primary value lies in capturing how postural balance deficits manifest during every day or sport-specific activities, thereby complementing instrumental measures that focus on the underlying mechanisms of postural control.

## 6. Postural Balance Across Populations and Functional Contexts

The manifestations, mechanisms, and consequences of postural balance impairments vary considerably across populations and real-life contexts. These differences reflect the influence of age, health status, physical condition, and task-specific demands on the underlying sensorimotor systems. In this section, we organize the evidence into four thematic domains to highlight how balance deficits emerge and are assessed in key populations: (1) aging and neurodegeneration; (2) pediatric and developmental considerations; (3) metabolic and musculoskeletal conditions; and (4) sport, performance, and injury.

The purpose of this section is not to provide an exhaustive clinical review of each condition, but rather to illustrate how postural balance is expressed and altered across representative populations and functional contexts. Detailed clinical analyses of specific disorders such as Parkinson’s disease, stroke, or multiple sclerosis are available in dedicated reviews and are beyond the scope of the present integrative framework.

### 6.1. Aging and Neurodegeneration

Age-related changes in postural balance are well documented and multifactorial, involving declines in sensory acuity (particularly proprioceptive and vestibular), motor function, and central processing [7,82]. Older adults typically show increased postural sway, delayed postural responses, and reduced use of anticipatory adjustments, especially under challenging sensory conditions (e.g., eyes closed, unstable surfaces) [83,84,85].

Balance deterioration with increasing age is a major contributor to fall risk, which represents a leading cause of injury, hospitalization, and mortality in this population [82,86]. Instrumental assessments frequently reveal increased center of pressure (COP) velocity and area, particularly in mediolateral sway, which is strongly linked to fall history and fear of falling. Clinical tests such as the Berg balance scale and timed up and go (TUG) test are widely used to quantify functional risk, although they may lack sensitivity in higher-functioning older adults [87].

In neurodegenerative conditions such as Parkinson’s disease, stroke, and multiple sclerosis, postural balance impairments are often more pronounced and complex [88,89]. In Parkinson’s disease, deficits arise from bradykinesia, rigidity, and altered central integration, leading to impaired APAs and reduced flexibility in postural strategy selection [90,91]. Poststroke individuals may present with asymmetric weight distribution, delayed muscle responses, and poor reactive balance control on the paretic side [92,93]. These deficits are associated with increased COP asymmetry, greater sway variability, and greater reliance on vision.

Together, aging and neurological conditions highlight the vulnerability of the balance system to multisystem decline. Assessment in these populations should include both instrumental evaluations (to quantify sensorimotor deficits) and functional tasks (to reflect real-world capacity), ideally under dual-task or perturbed conditions, to challenge compensatory mechanisms.

### 6.2. Pediatric and Developmental Considerations

In children and adolescents, postural balance reflects the maturational trajectory of the sensory and neuromuscular systems. Compared with older peers or adults, young children typically exhibit greater sway and less consistent balance strategies due to immature proprioception, incomplete sensory integration, and the development of coordination [30,94].

During normal development, improvements in balance coincide with increased sensorimotor sophistication, including better reweighting of visual and proprioceptive cues. However, in conditions such as developmental coordination disorders, attention-deficit/hyperactivity disorders, or cerebral palsy, these processes may be delayed or impaired. For example, children with cerebral palsy often rely excessively on visual input and show reduced ankle strategy use, leading to decreased postural balance [95,96].

Assessment tools in pediatric populations must be age-appropriate and sensitive to developmental norms. Clinical tests such as the Pediatric Balance Scale or tandem stance are useful, whereas posturographic evaluations (force platforms) can reveal subtle deficits in sensory reweighting or strategy selection. Interventions focused on enhancing sensory integration and motor planning have shown promise in improving balance control in children with motor impairments.

### 6.3. Metabolic and Musculoskeletal Conditions

Obesity and metabolic disorders are increasingly recognized as influential factors in postural balance across both pediatric and adult populations. Excess body mass alters the distribution of the center of mass, increases postural sway, and impairs proprioceptive feedback, especially from the lower limbs. Individuals with obesity often exhibit increased COP displacement and velocity, particularly in the mediolateral direction, and show reduced balance performance in both static and dynamic tasks [4,97].

Mechanistically, increased adiposity is thought to affect balance through reduced plantar sensitivity, delayed muscle activation, and altered joint kinematics [98,99,100]. In children, these changes may also delay the development of mature balance strategies, compounding functional limitations. Both clinical assessments and posturography reveal consistent deficits, although tailored interventions targeting strength, coordination, and proprioception are effective in mitigating risk [101].

In musculoskeletal disorders, such as chronic ankle instability, anterior cruciate ligament (ACL) reconstruction, or chronic low back pain, balance impairments are linked to proprioceptive deficits, muscle inhibition, and altered neuromuscular control. For example, individuals with chronic ankle instability often show decreased dynamic balance in the affected limb, particularly in single-leg stances and reach tasks [102,103]. Postural asymmetry and a reduced ability to recover from perturbations are common findings.

Instrumental assessments frequently demonstrate side-to-side differences in sway patterns or COP metrics, whereas functional tests such as the SEBT or Y-Balance Test are commonly used to evaluate dynamic balance in musculoskeletal populations [77,78]. Targeted rehabilitation, including neuromuscular re-education, balance training, and sensorimotor feedback, has been shown to restore postural control and reduce reinjury rates.

### 6.4. Sports, Performance, and Injury Prevention

In athletic populations, postural balance plays a dual role: it supports optimal performance and serves as a protective mechanism against injury. Compared with nonathletes, high-level athletes typically exhibit superior balance control, particularly in tasks requiring dynamic postural adjustments, rapid directional changes, or dual-task coordination [104,105,106]. However, fatigue, overtraining, or neuromuscular deficits can compromise this advantage. For example, impaired balance has been linked to an increased risk of lower limb injuries, such as ACL tears or ankle sprains [107,108,109].

Postural balance can also be trained and enhanced through targeted interventions, including proprioceptive training, unstable surface exercises, and neuromuscular conditioning [81,110]. Improvements in balance have been associated with better agility, greater strength symmetry, and decreased injury incidence across sports.

## 7. Functional and Clinical Implications of Postural Balance

Postural balance is not only a marker of sensorimotor integrity but also a foundational determinant of functional independence, motor efficiency, and injury prevention. Its impairment has significant clinical and functional consequences across the lifespan, from delayed motor development in children to increased fall risk in older adults. This section synthesizes the broader implications of postural balance, highlighting its role as a target for assessment, intervention, and functional enhancement in diverse health and performance contexts.

### 7.1. Postural Balance and Functional Independence

The ability to maintain postural balance is essential for executing basic and instrumental activities of daily living. Tasks such as rising from a chair, climbing stairs, dressing, or navigating crowded environments all require continuous postural adjustments to maintain stability during voluntary movement [21,54]. Deficits in balance often lead to activity limitations, reduced mobility, and avoidance of challenging situations due to fear of falling, ultimately contributing to a decline in functional independence [111].

In clinical settings, postural balance is a key component of functional assessment batteries and a predictor of mobility outcomes, hospitalization, and institutionalization [67,112]. Rehabilitation programs targeting balance, particularly through task specific, progressive, and multisensory training, can significantly improve autonomy and quality of life in older adults and individuals with chronic conditions [113,114,115].

### 7.2. Balance Impairments and Fall Risk

Among the most widely recognized consequences of impaired postural balance is an elevated risk of falls. Falls represent a major public health issue, especially in the aging population, and are associated with fractures, head injuries, fear of falling, and premature mortality. Importantly, many falls are preventable, and postural balance is one of the most modifiable risk factors [82].

Poor performance on balance tests (e.g., high COP sway velocity, unstable single-leg stance, low functional reach) is strongly associated with both retrospective and prospective fall incidence [116,117,118]. Screening for balance impairments is therefore critical for the early identification of individuals at risk. Multimodal interventions combining balance, strength, and cognitive–motor training have proven effective in reducing fall frequency and improving postural control [119,120].

### 7.3. Postural Balance and Motor Performance

Postural balance underpins not only stability but also movement precision, speed, and coordination. Effective balance control minimizes unnecessary muscular cocontraction, allows for smoother transitions between postures, and supports efficient force production during locomotion and task execution [121,122].

In athletic populations, balance capacity is closely linked to performance metrics such as agility, jumping mechanics, and directional changes [79,110]. This relationship can be attributed to sport-specific adaptations in sensorimotor control as evidenced by studies showing that targeted neuromuscular interventions consistently improve dynamic balance in athletes [81,110]. For instance, elite athletes, such as top-level volleyball players, exhibit postural strategies characterized by more precise sway regulation, superior postural stability under perturbation, and higher movement complexity, reflecting an optimized, automated integration of balance control that supports motor performance [104]. Conversely, impaired balance is associated with delayed motor responses, reduced movement economy, and a greater likelihood of compensatory strategies that compromise technique and increase injury risk [123,124].

Training interventions that enhance sensorimotor integration, proprioceptive acuity, and dynamic stability contribute to improved motor performance, especially in sports requiring complex whole-body coordination. This principle also applies to rehabilitation and neurorehabilitation, where restoring balance capacity facilitates the recovery of gait and functional movement.

### 7.4. Clinical Relevance of Balance Assessment and Training

Given its broad impact on health and function, postural balance should be routinely assessed in both clinical and performance settings. However, effective application requires careful consideration of the tools and protocols used. As outlined earlier, no single test captures the full complexity of balance; rather, a combination of instrumental, functional, and task-specific assessments provides a more comprehensive picture.

Balance training should be individualized, progressive, and context specific. Compared with static or unidimensional approaches, programs incorporating variable sensory conditions, dual-task challenges, and dynamic perturbations have been shown to elicit greater adaptations. Moreover, the transfer of balance improvements to functional outcomes depends on the ecological validity of the training tasks.

Beyond rehabilitation, balance enhancement is increasingly integrated into preventive strategies, for example, in fall prevention programs for older adults, injury prevention in athletes, or motor skill development in children. As such, postural balance represents not only a rehabilitative goal but also a modifiable determinant of long-term functional capacity.

## 8. Conclusions

Postural balance is a complex motor skill emerging from the dynamic interaction of sensory inputs, neural processing, and neuromuscular responses that together regulate the position and movement of the body in space. Rather than being governed by a single system, balance reflects the coordinated activity of multiple sensorimotor mechanisms operating within task, environmental, and individual constraints. Understanding these mechanisms is essential for interpreting balance behavior across different contexts and populations.

The integrative framework presented in this review highlights how postural balance can be understood as an emergent functional outcome of distributed postural control processes. By linking mechanistic perspectives, assessment strategies, and population-specific manifestations, this framework contributes to a more comprehensive interpretation of balance as a dynamic and context-dependent motor capability relevant to health, rehabilitation, and human performance.

Future research should aim to address several key gaps in the current literature. First, there is a need for more integrative assessment protocols that combine instrumental posturography with functional balance tests in order to better link underlying postural control mechanisms with real-world performance outcomes. Second, greater methodological standardization in force-platform protocols, including sampling frequency, trial duration, and task conditions, is necessary to improve comparability across studies. Third, further investigation of cognitive–motor interactions under ecologically valid dual-task conditions may help clarify how attentional demands influence balance control in both healthy and clinical populations. Finally, longitudinal and developmental studies examining how postural control mechanisms evolve across the lifespan and across different health conditions would contribute to a more comprehensive understanding of balance as an adaptive sensorimotor function.

Future research may also benefit from the incorporation of emerging technologies that expand the possibilities for balance assessment beyond traditional laboratory settings. Wearable inertial sensors, for example, allow the continuous monitoring of postural stability during daily activities and functional tasks, providing ecologically valid information about balance performance. In addition, advances in machine learning and signal processing techniques may enable more sophisticated analyses of postural sway dynamics, helping to identify subtle patterns of balance impairment that may not be detectable using conventional metrics. The integration of these technological approaches could contribute to more precise, accessible, and context-sensitive assessment strategies for postural balance in both research and clinical practice.

## Figures and Tables

**Table 1:** Sensory systems involved in postural control: functional roles and implications for postural balance.

	Visual System	Vestibular System	Somatosensory System
**Main Receptors and Anatomical Structures**	Photoreceptors (rods and cones) in the retina; optic nerve; primary and associative visual cortices.	Semicircular canals (angular acceleration); otolith organs (utricle and saccule: linear acceleration and gravito-inertial forces); vestibular nuclei and vestibulospinal pathways.	Proprioceptors: muscle spindles (muscle length and velocity), Golgi tendon organs (muscle tension), joint capsule and ligament receptors. Cutaneous mechanoreceptors: primarily located on the plantar surface of the feet (pressure, vibration, skin stretch).
**Type of Information Provided**	Optic flow (visual field motion); spatial orientation cues relative to the external environment (verticality and horizon); depth perception.	Head motion and orientation relative to gravity; angular velocity and linear acceleration of the head.	Proprioceptive input: joint position sense, movement sensation (kinaesthesia), and muscle tension gradients across the kinematic chain. Cutaneous input: information about the support surface interface, center of pressure displacement, and shear forces.
**Primary Functional Role in Postural Control**	Provides an exocentric reference frame for regulating body sway; supports anticipatory postural adjustments through continuous environmental scanning.	Provides a gravity-referenced (geocentric) signal; resolves sensory conflicts between visual and somatosensory inputs; contributes to rapid vestibulospinal reflexes for postural stabilization.	Provides an egocentric reference frame for segmental alignment relative to the base of support; mediates rapid reflexive and automatic postural responses to perturbations.
**Conditions or Tasks in Which the System Is Particularly Relevant**	Quiet stance in well-lit and stable environments; navigation in complex or dynamic surroundings; tasks requiring precise alignment with external references.	Dynamic head movements; standing or walking on unstable or tilting surfaces; environments with reduced or absent visual information (e.g., darkness or eyes-closed conditions).	Upright stance on firm and stable surfaces; detection of subtle body sway during quiet standing; early phase of reactive responses to sudden perturbations.
**Implications for Postural Balance Performance**	Plays a major stabilizing role when somatosensory or vestibular inputs are unreliable. Excessive visual dependence may increase instability under visual conflict or moving visual environments.	Essential for maintaining postural orientation and distinguishing self-motion from environmental motion. Vestibular dysfunction markedly impairs balance, particularly during dynamic tasks or when vision is unavailable.	Serves as the primary modality for baseline postural control on stable surfaces. Reduced somatosensory acuity (e.g., peripheral neuropathy or joint injury) impairs balance and necessitates compensatory sensory reweighting toward visual or vestibular reliance.

**Table 2:** Commonly used clinical and functional tests for the assessment of postural balance across populations.

Population	Test	Type of Balance Assessed	Main Characteristics	Typical Purpose
Older adults	Berg Balance Scale (BBS)	Static and dynamic balance	Multitask scale including transfers, reaching, turning, and standing tasks	Global balance assessment; fall risk screening
	Timed Up and Go (TUG)	Functional balance and mobility	Timed task including sit-to-stand, walking, turning, and sitting	Screening of mobility limitations and fall risk
	Tinetti Performance-Oriented Mobility Assessment	Balance and gait	Combined evaluation of standing balance and gait characteristics	Identification of balance and gait impairments
	Single-leg stance	Static balance	Time-based unipedal stance task	Screening of postural stability and fall risk
Pediatric population (preschool and early school age)	Pediatric Balance Scale	Static and dynamic balance	Adapted version of BBS for children	Assessment of basic postural stability
	Single-leg stance	Static balance	Age-adjusted unipedal stance task	Evaluation of emerging balance control
	Tandem stance	Static balance	Narrow base-of-support stance	Screening of postural stability
Older children and adolescents	Tandem walking/line walking	Dynamic balance	Gait task with reduced base of support	Evaluation of dynamic postural control
	Functional motor batteries (e.g., BOT-2 balance items)	Static and dynamic balance	Standardized motor proficiency tasks	Assessment of balance within global motor competence
	Star Excursion Balance Test (SEBT)/Y-Balance Test	Dynamic balance	Multidirectional reach task in single-leg stance	Screening of dynamic balance deficits
General adult population	Single-leg stance (EO/EC)	Static balance	Unipedal stance with sensory manipulation	Screening of postural stability
	Tandem stance	Static balance	Reduced base-of-support stance	Detection of subtle balance impairments
	Functional Reach Test	Dynamic balance	Forward reach while standing	Evaluation of voluntary control of center of mass
	SEBT/Y-Balance Test (active adults)	Dynamic balance	High demand unipedal dynamic tasks	Detection of subtle postural control deficits
Athletic populations	SEBT/Y-Balance Test	Dynamic balance	Unipedal multidirectional reach task assessing neuromuscular control, limits of stability, and limb asymmetry	Injury risk screening, rehabilitation monitoring, and return-to-play decision-making

BBS, Berg Balance Scale; TUG, Timed Up and Go; SEBT, Star Excursion Balance Test; EO, eyes open; EC, eyes closed; BOT-2, Bruininks–Oseretsky Test of Motor Proficiency, Second Edition.

## Data Availability

No new data were created or analyzed in this study. Data sharing is not applicable to this article.

## Abbreviations

The following abbreviations are used in this manuscript:

ACL	Anterior Cruciate Ligament
APAs	Anticipatory Postural Adjustments
BBS	Berg Balance Scale
BOT-2	Bruininks–Oseretsky Test of Motor Proficiency, Second Edition
COP	Center of Pressure
RMS	Root Mean Square
SEBT	Star Excursion Balance Test
TUG	Timed Up and Go
